# Densfraktur nach Hochrasanztrauma: Instabilität richtig einschätzen

**DOI:** 10.1007/s00113-021-01062-y

**Published:** 2021-08-05

**Authors:** K. Hemker, M. Stangenberg, M. Dreimann, L. Köpke, A. Heuer, L. Viezens

**Affiliations:** Klinik und Poliklinik für Unfallchirurgie und Orthopädie, Universitätsklinikum Hamburg-Eppendorf, Martinistraße 52, 20251 Hamburg, Deutschland

**Keywords:** Wirbelfraktur, Halswirbelsäulenverletzung, Myelopathie, Instabilität, Polytrauma, Vertebral fracture, Cervical spine injury, Myelopathy, Instability, Polytrauma

## Abstract

Densfrakturen sind häufige Verletzungen der Halswirbelsäule und kommen meist in höherem Lebensalter vor; hierbei sind diese oft durch Bagatelltraumata bedingt. Bei jüngeren Patienten werden diese v. a. im Rahmen von Hochrasanztraumata beobachtet. Klassifiziert werden die Densfrakturen nach Anderson und D’Alonzo. Selten kommt es durch die Fraktur zu einer zervikalen Myelopathie, die lebensbedrohlich sein kann.

In diesem Artikel werden zwei Fälle von Patienten mit Densfrakturen mit traumatischer Myelopathie dargestellt. Beim ersten Fall handelt es sich um eine Typ-III-Fraktur, beim anderen Fall um eine Typ-II-Fraktur. In beiden Fällen wurde die vorliegende Instabilität aufgrund der anatomischen Stellung in der initialen Computertomographie (CT) falsch eingeschätzt. Im weiteren Verlauf zeigte sich in beiden Fällen eine erhebliche Instabilität, aufgrund deren es zu fatalen Myelonverletzungen gekommen war.

In diesem „case report“ soll auf das mögliche Vorliegen von Myelonverletzungen bei vermeintlich trivialen Densfrakturen bei stattgehabten Hochrasanztraumata aufmerksam gemacht werden. Insbesondere bei reanimationspflichtigen Patienten ohne internistische Ursache muss an eine Myelonkompression gedacht werden. Sollte der Patient bei Vorliegen einer knöchernen Verletzung im CT z. B. durch eine Intubation klinisch nicht ausreichend beurteilbar sein, muss die Indikation zur Magnetresonanztomographie großzügig gestellt werden. Nur durch diese wird einem die frühzeitige Erkennung einer Myelopathie und die rechtzeitige Therapie ermöglicht.

## Abstract (en)

Odontoid process fractures are frequent injuries of the cervical spine and usually occur at an advanced age and often due to minor trauma. In younger patients they are mainly observed in the context of high impact trauma. Odontoid fractures are classified according to Anderson and D’Alonzo. In rare cases the fracture results in cervical myelopathy, which can be life-threatening.

In this article, two cases of patients with odontoid fractures and traumatic myelopathy are presented. The first case concerns a type III fracture, the second a type II fracture. In both cases the instability present was incorrectly assessed on the basis of the anatomical position in the initial computed tomography (CT). In the further course both cases showed considerable instability, which led to fatal spinal cord injuries.

The purpose of this case report is to draw attention to the possible presence of spinal cord injuries in supposedly trivial odontoid fractures when high speed trauma has occurred. In particular, spinal cord compression should be considered in patients requiring resuscitation without having an internal medical cause. If the patient with a bone injury on CT, e.g. cannot be adequately assessed clinically by intubation, the indications for magnetic resonance imaging must be generously considered. This is the only way to ensure early detection of a myelopathy and timely treatment.

## Einleitung

Bei Verletzungen der Halswirbelsäule ist in einem Drittel der Fälle die obere Halswirbelsäule betroffen. Neben der Densfraktur, Atlasfraktur, Okzipitalkondylenfraktur und der „hangman’s fracture“ sind auch atlantookzipitale und atlantoaxiale ligamentäre Verletzungen beschrieben. Bei den Densfrakturen handelt es sich mit ca. 25 % aller HWS-Frakturen um die häufigsten Verletzungen der Halswirbelsäule mit einer steigenden Inzidenz mit zunehmendem Lebensalter. Oft sind diese Hyperextensionsverletzungen im geriatrischen Patientenkollektiv im Rahmen von Bagatelltraumata [10, 12, 15].

**Figure 1:**
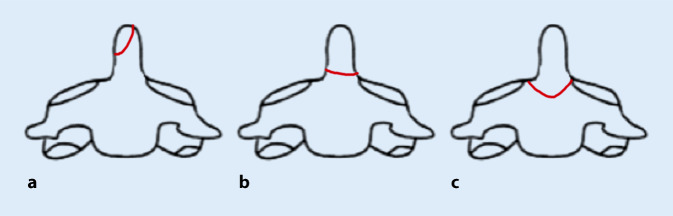

Die gängigste Klassifikation der Densfrakturen ist die von Anderson und D’Alonzo. Hierbei werden 3 Typen unterschieden. Typ-I-Frakturen betreffen die Densspitze, Typ-II-Frakturen den Übergang vom Dens zum Corpus und Typ-III-Frakturen die Densbasis (Abb. 1; [1]). Typ-I- und Typ-III-Frakturen werden in der Regel als stabile Frakturen bewertet und der konservativen Therapie zugeführt. Bei Typ-II-Frakturen sind die Empfehlungen uneinheitlich; diese werden im deutschen Sprachraum jedoch häufig als instabil gewertet und der operativen Therapie zugeführt. Alternativ ist bei diesen Frakturen auch eine konservative Therapie mittels Zervikalorthese möglich [12].

Bei jüngeren Patienten treten Densfrakturen häufig im Rahmen von Hochrasanztraumata auf. Bislang sind Densfrakturen mit primärer Myelopathie in der Literatur nur in geringer Anzahl beschrieben [6, 14, 17].

Anhand von zwei Fallbeispielen soll die Fehleinschätzung traumatischer Densfrakturen aufgezeigt werden, bei denen es sich gerade nach Hochrasanztraumata um instabile Verletzungen im Sinne von B‑Verletzungen handeln kann und bei denen die zerrissenen Bandstrukturen radiologisch nicht adäquat dargestellt werden können.

## Fallbeschreibung 1

**Figure 2:**
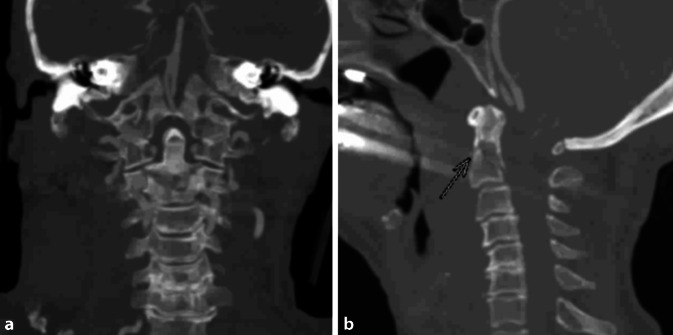

Die 76-jährige Patientin wurde nach einem Frontalzusammenstoß mit ihrem Pkw gegen einen ca. 70 km/h fahrenden Lkw in ein nahegelegenes Klinikum eingeliefert. Initial sei die Patientin bewusstlos gewesen. Es erfolgte eine konventionelle Röntgendiagnostik, welche eine alte Beckenfraktur, eine Densfraktur und eine Tibiafraktur rechts zeigte. Aufgrund der Verletzungsschwere erfolgte die Sekundärverlegung in ein Level-1-Traumazentrum. Bei Ankunft im Schockraum erfolgte die Behandlung gemäß ATLS-Empfehlung. Die Patientin war A‑, B‑, C‑ und D-stabil; der GCS lag bei 15. Die FAST zeigte keine freie intraabdominelle Flüssigkeit. Der Stiffneck war seit dem Unfallort anliegend. Es zeigte sich in der Untersuchung ein Druckschmerz über der Halswirbelsäule. Darüber hinaus zeigten sich ein Thoraxkompressionsschmerz, Hämatome über der linken Klavikula, dem linken Oberarm sowie prätibial beidseits und eine Schwellung des rechten Unterschenkels. Die periphere Durchblutung, Motorik und Sensibilität waren intakt. Zudem war bei im Röntgen diagnostizierter Beckenfraktur ein „pelvic binder“ anliegend. In der durchgeführten Traumaspirale mit Gefäßdarstellung zeigten sich eine nichtdislozierte Densfraktur Typ III nach Anderson und D’Alonzo (Abb. 2), Rippenfrakturen beidseits, eine laterale Klavikulafraktur links (AO 15-C1.2), eine Olekranonfraktur links (AO 13-B2.2), eine Unterarmprellung rechts, eine proximale Unterschenkelfraktur rechts (AO 41-B2) sowie eine Os-metatarsale-V-Schaftfraktur rechts. Intrakranielle Traumafolgen konnten ausgeschlossen werden, sodass bei initialer Bewusstlosigkeit von einer Commotio cerebri ausgegangen wurde. Vorbekannt waren ein bradyarrhythmisches Vorhofflimmern; die Patientin hatte aufgrund dessen einen Herzschrittmacher und war unter Eliquis antikoaguliert.

Bei Polytrauma mit nichtdislozierter Densfraktur Typ III nach Anderson und D’Alonzo wurde sich für die Ruhigstellung der Halswirbelsäule mittels Philadelphia-Orthese entschieden. Funktionsaufnahmen unter Durchleuchtung zur Überprüfung der Stabilität sollten im Verlauf erfolgen.

Bei respiratorischer Insuffizienz wurde die Patientin noch am Aufnahmetag bei dem Verdacht auf eine stattgehabte Aspiration während der initialen Bewusstlosigkeit intubiert und auf die Intensivstation verlegt. Trotz antibiotischer Therapie mit Meropenem entwickelte sich eine ausgeprägte hämodynamische Instabilität am ehesten aufgrund einer Aspirationspneumonie, die eine hochdosierte Katecholamintherapie erforderlich machte.

**Figure 3:**
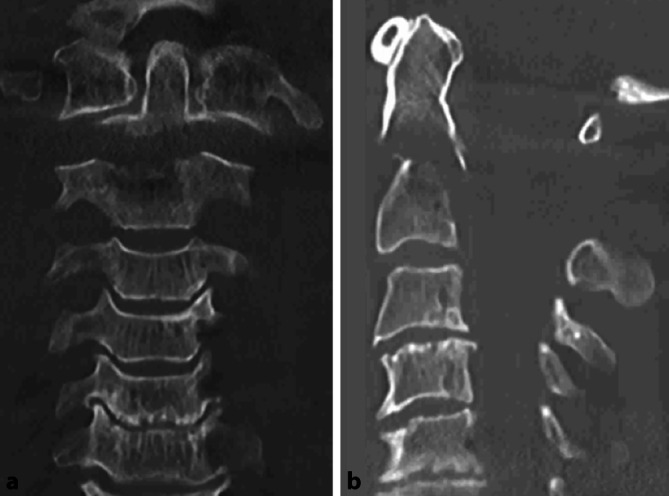

Aufgrund einer hämodynamisch relevanten Tachykardie kam es zu einer kurz andauernden Reanimation mit Dislokation des Beatmungstubus, was bei erfolgloser Reintubation durch nichtmögliche Reklination des Kopfes eine Nottracheotomie erforderlich machte. Hierfür wurde die Zervikalorthese kurzzeitig entfernt, sodass am Folgetag eine Verlaufscomputertomographie durchgeführt wurde. In dieser zeigte sich eine Dislokation der Densfraktur mit Distraktion um mehr als 1 cm (Abb. 3) ohne Anhalt auf eine Myeloneinklemmung, welche in der Gesamtsituation bei Ausbleiben von Spontanatmung und vermutetem hohen Querschnitt interdisziplinär als nichtüberlebbar eingestuft wurde. Im Konsens mit den Angehörigen wurden die intensivmedizinischen Maßnahmen beendet, sodass die Patientin unter Analgosedierung 7 Tage nach dem Unfall verstarb.

## Fallbeschreibung 2

**Figure 4:**
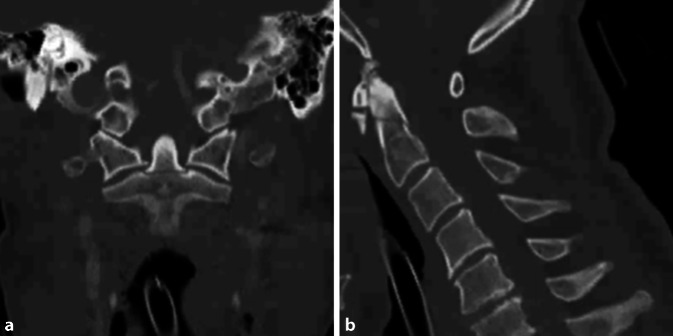

Ein 57-jähriger Patient wurde nach einem Hochrasanztrauma mit Frontalzusammenstoß als Fahrer eines Pkw in den Schockraum eingeliefert. Nach einer Crash-Rettung kam es zu einer 3‑minütigen Reanimation des Verunfallten im Rettungswagen. Der Patient zeigte zuvor weite lichtstarre Pupillen, nach der Reanimation verengte lichtstarre Pupillen. Vorerkrankungen waren nicht bekannt. Bei der Untersuchung im Schockraum zeigten sich keine äußerlichen Verletzungen. Außerdem zeigte sich die gesamte Wirbelsäule ohne Stufenbildung; auch in der FAST zeigte sich kein Anhalt auf freie intraabdominelle Flüssigkeit. In der Traumaspirale stellte sich eine nichtdislozierte Densfraktur Typ II nach Anderson D’Alonzo ohne Einengung des Spinalkanals dar (Abb. 4). Darüber hinaus präsentierten sich bilaterale Infiltrate der Lungenunterlappen am ehesten nach Aspiration.

Zur weiteren Ruhigstellung erfolgte der Austausch des Stiffneck gegen eine Philadelphia-Orthese, um Weichteilkomplikationen zu vermeiden. Bei rezidivierender Kreislaufinstabilität und zur Planung einer operativen Versorgung erfolgte 2 Tage später die erweiterte Bildgebung mittels MRT, mit der Frage nach einer diskoligamentären Beteiligung sowie einer Myelonbeteiligung.

**Figure 5:**
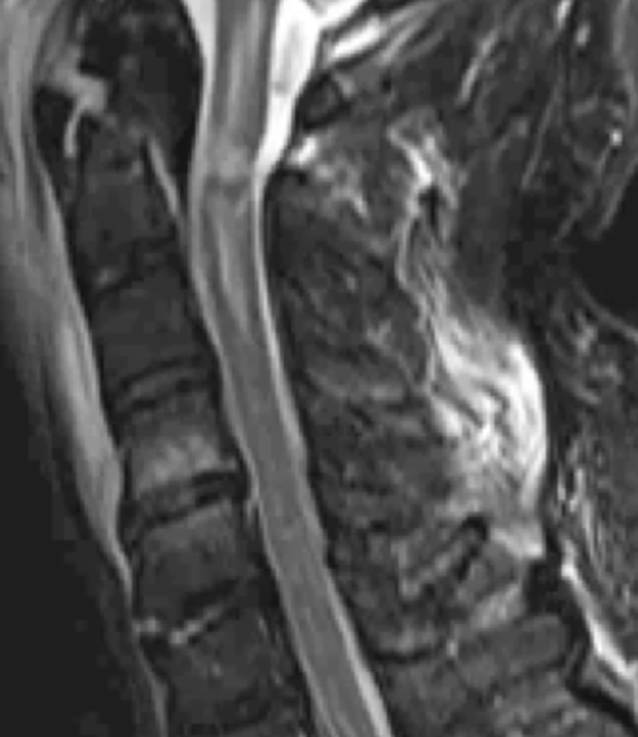

Hier präsentierte sich nun eine sekundäre Dislokation der bekannten Densfraktur Typ II nach Anderson und D’Alonzo nach dorsal. Ebenso zeigte sich eine deutliche Myelonkontusion auf Höhe der Densfraktur mit einem Ödem vom Hirnstamm bis zum HWK 3 reichend. Darüber hinaus zeigte sich eine diskoligamentäre Zerreißung auf Höhe HWK 5/6 und HWK 7/BWK 1 (Abb. 5).

**Figure 6:**
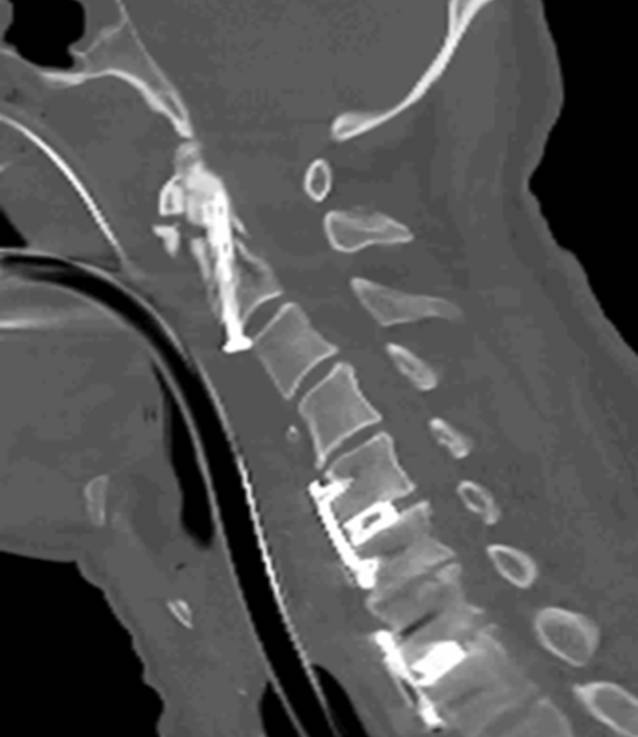

Nach 3 Tagen unter leitliniengerechter Hypothermiebehandlung und zwischenzeitiger rezidivierender Kreislaufinstabilität, welche eine frühere Operation unmöglich machte, erfolgte die operative Versorgung der Densfraktur nach Böhler [3, 16]. Ebenso wurden eine anteriore Diskektomie und Fusion mit „cage“ und Platte auf den Höhen HWK 5/6 und HWK 7/BWK 1 durchgeführt (Abb. 6). Um eine weitere Manipulation des Myelons zu vermeiden, wurde nach der ventralen Stabilisierung bei ausreichendem Liquorreserveraum auf eine weitere Dekompression und Stabilisierung von dorsal verzichtet.

Im weiteren Verlauf bestand eine durchgehende Sinusbradykardie, die bei zweimaligem Sinusarrest zu jeweiliger medikamentöser Reanimationspflichtigkeit führte. In den kraniellen Verlaufsbildgebungen zeigte sich weiterhin eine progrediente Myelopathie. Bei „locked-in syndrome“ mit zu erwartendem hohem Querschnitt und voraussichtlicher dauerhafter Beatmungspflichtigkeit erfolgte im Konsens gemeinsam mit den Angehörigen und im mutmaßlichen Patientenwillen die Entscheidung für eine palliativ-supportive Therapie, sodass der Verunfallte 5 Tage nach dem Unfall verstarb.

## Diskussion

Degenerative zervikale Myelopathien stellen den Großteil der zervikalen Myelopathien dar [4]. Traumatische zervikale Myelopathien sind hingegen selten und häufig durch B‑Verletzungen im Rahmen von Hyperextensionstraumata bedingt. Nicht selten sind erworbene zervikale Stenosen und Spondylosen begünstigende Faktoren einer traumatischen Myelopathie. Typischerweise zeigen sich die zervikalen Myelopathien in motorischen Ausfällen der Arme und Beine, als Gangataxien und Sensibilitätsstörungen [7, 9, 17]. In der Bildgebung stellt sich eine Myelopathie als Hyperintensität des Myelons in der T2-Wichtung dar, bei traumatischer Genese sieht man häufig begleitend ein Hämatom oder eine diskoligamentäre Verletzung. Oft präsentiert sich dies auch ohne Vorliegen von Frakturen [9]. Die frühzeitige und sichere Diagnosestellung von Verletzungen der oberen Halswirbelsäule ist im Hinblick auf die Therapieentscheidung und die Vermeidung von Spätfolgen besonders relevant [5, 14, 16].

In beiden Fallbeispielen kam es innerhalb einer Woche nach den Hyperextensionstraumata der HWS im Rahmen von Hochrasanztraumata zum Exitus letalis durch ein hoch zervikales Myelonödem bei instabilen Densfrakturen.

Neurologische Ausfälle bei Densfrakturen treten insgesamt nur selten auf. Dies ist auf den weiten Durchmesser des Spinalkanals von etwa 22 mm auf Denshöhe bei einem Myelondurchmesser von etwa 10 mm zurückzuführen [7, 14]. Bei 6–25 % der Patienten mit Densfrakturen kommt es zum Auftreten von neurologischen Ausfällen, wie z. B. Armschwächen, okzipitaler Hypästhesie, Hyperreflexie der Beine, Paraparesen oder dem Auftreten eines Brown-Séquard-Syndroms [1]. Bei 75 % dieser zeigt sich schubweise eine neurologische Verschlechterung [7]. Über die Hälfte der Patienten zeigen nach der Frakturversorgung jedoch eine Remission [1].

Beachtet werden muss, dass bei nichtbeurteilbaren Patienten mit fraglich instabilen Verletzungen der Halswirbelsäule eine schonende Intubation erfolgen soll. Hierbei kommen am ehesten nach der S3-Leitlinien für Polytrauma‑/Schockraumpatienten eine fiberoptische Intubation sowie die manuelle „In-line“-Stabilisation in Betracht [2].

Unterschiede zwischen konventioneller Intubation und einem Larynxtubus bestehen bei instabilen Halswirbelsäulenverletzung jedoch nicht [18]. Im Fall 1 erfolgte eine fiberoptische Intubation, im zweiten Fall ist die Intubation am Unfallort erfolgt und unbekannt.

Initial wird bei Verdacht auf Densfrakturen eine Röntgenaufnahme der Halswirbelsäule in zwei Ebenen mit Dens-Zielaufnahme durchgeführt. Nach der Leitlinie der Deutschen Gesellschaft für Unfallchirurgie wird bei polytraumatisierten und bewusstlosen Patienten bei Verletzungen der oberen Halswirbelsäule frühzeitig ergänzend zum Röntgen eine radiologische Diagnostik mittels CT empfohlen [5, 13]. Die empfohlene Diagnostik haben wir in beiden Fällen durchgeführt. Die Verlaufsformen der beiden Densfrakturen zeigten sich ähnlich. Die Fraktur von Fall 1 wurde als Typ III nach Anderson und D’Alonzo klassifiziert, die von Fall 2 als Typ-II-Fraktur. Beide Frakturen wurden initial als stabil eingeschätzt.

Im ersten Fall erfolgte eine leitliniengerechte konservative Versorgung mittels semirigider Orthese [11, 13]. Trotz Ruhigstellung kam es sekundär zu einer Frakturdislokation um etwa 1 cm nach kranial und im Rahmen dieser zu einer Distraktion des Myelons, welche zu kardialen Rhythmusstörungen mit Reanimationspflichtigkeit führte.

Ebenso erfolgte im zweiten Fall bei einer in der initialen Bildgebung nichtdislozierten Typ-II-Fraktur nach Anderson und D’Alonzo die Ruhigstellung in einer semirigiden Orthese, und es wurde die Indikation zur konservativen Therapie gestellt.

Bei rezidivierender Kreislaufinstabilität des Patienten erfolgte eine MRT der Halswirbelsäule, in der sich eine Frakturdislokation mit Myelopathie zeigte und die Indikation zur Operation gestellt wurde. Intraoperativ zeigte sich eine extreme Instabilität der Fraktur; die ventrale Osteosynthese erfolgte nach Böhler.

Die Therapie der Densfrakturen ist nicht immer eindeutig. Insbesondere bei Typ-II-Frakturen kann sich die optimale Therapiefindung schwierig darstellen.

**Figure 7:**
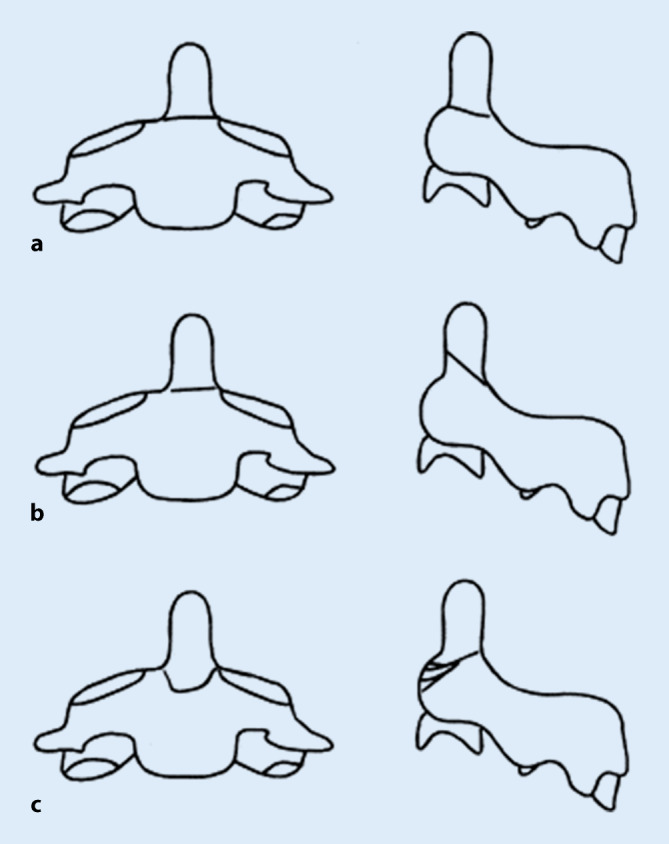

Verschiedene Autoren, wie Eysel und Roosen 1993 [8] und Grauer et al. 2005 [9], haben eine weitere Einteilung der Typ-II-Frakturen nach Anderson und D’Alonzo vorgenommen, um das operative Vorgehen festzulegen. Bei beiden Publikationen wird die Typ-II-Fraktur nach Anderson und D’Alonzo in 3 Subtypen gegliedert und daraus die Therapieempfehlung abgeleitet (Abb. 7).

Bei Subtyp A, ohne Auftreten einer Dislokation, ist eine äußere Immobilisation mittels Zervikalorthese empfohlen. Bei Vorliegen einer transversalen Dislokation oder eines von anterior-superior nach posterior-inferior verlaufenden Frakturspalts spricht man vom Subtyp B. In diesem Fall wird eine anteriore Schraubenfixation empfohlen. Bei einer eingestauchten Fraktur oder einem Frakturverlauf von anterior-inferior nach posterior-superior liegt der Subtyp C vor. In diesem Fall soll eine posteriore atlantoaxiale Fusion von HWK 1 und 2 erfolgen [7].

Bei neurologisch unauffälligem, wachem und orientiertem Patienten wird bei CT-morphologischem Verdacht auf eine diskoligamentäre Instabilität nach Leitlinien der DGU eine dynamische Röntgenuntersuchung empfohlen. Im Falle eines posttraumatisch CT-morphologisch unerklärten neurologischen Defizites wird unverzüglich zur MRT-Untersuchung geraten [13]. In beiden Fallbeispielen waren die Patienten intubiert und beatmet. Eine adäquate neurologische Untersuchung mit Überprüfung der Sensomotorik und Kraftgrade war daher nicht möglich. Das Vorliegen von neurologischen Ausfällen konnte anhand der Klinik demnach nicht ausgemacht werden. In beiden Fällen stellte sich nach den Leitlinien somit weder die Indikation der dynamischen Röntgenuntersuchung noch der sofortigen Durchführung einer MRT.

Im zweiten Fallbeispiel führten wir bei Verdacht auf eine Myelopathie bei instabiler Densfraktur eine MRT der HWS durch, in welcher sich eine deutliche Myelonkontusion auf Höhe der Densfraktur mit einem ausgeprägten Myelonödem und nebenbefundlich eine diskoligamentäre Zerreißung zeigten.

In einer amerikanischen „Single-center“-Studie, die über 6000 Patienten mit stumpfem Kopftrauma untersuchte, zeigten sich bei etwa 5 % der Patienten, die ein zervikales CT erhielten und noch Druckschmerzen im Bereich der Halswirbelsäule empfanden, im MRT signifikante Befunde im Vergleich zum CT, die das Therapievorgehen veränderten [6].

Auch wenn es nach der CT-Bildgebung eine stabile Densfraktur zu sein scheint, sollte beachtet werden, dass es sich auch um eine B‑Verletzung mit Ruptur der dorsalen Strukturen handeln kann.

Bei Densfrakturen im Rahmen von Hochrasanztraumata sollte daher niederschwellig ein zervikales MRT durchgeführt werden, um eine Myelopathie im Rahmen einer B‑Verletzung frühzeitig zu entdecken und das Therapievorgehen anzupassen.

## Fazit für die Praxis

Verletzungen der oberen HWS sind in der Regel einfache Frakturen ohne höhergradige Instabilität, regelhaft im geriatrischen Patientenkollektiv.

Sollten diese jedoch im Rahmen von Hochrasanztraumata auftreten, muss man immer an das Vorliegen von komplexeren Verletzungen mit bestehender Instabilität denken. Insbesondere bei Reanimationspflichtigkeit ohne internistische Gründe muss an eine B‑Verletzung mit Kompression des Myelons gedacht werden.

Sollte eine knöcherne Verletzung im CT nach Hochrasanztrauma sichtbar und eine klinische Beurteilung des Patienten z. B. im Rahmen einer Intubation nicht möglich sein, sollte auch bei nichtdislozierten Frakturen niederschwellig eine MRT durchgeführt werden. Nur so kann eine Myelonverletzung erkannt und ggf. noch rechtzeitig therapiert werden.
